# Effectiveness of clinical breast examination as a ‘stand-alone’ screening modality: an overview of systematic reviews

**DOI:** 10.1186/s12885-020-07521-w

**Published:** 2020-11-09

**Authors:** Tran Thu Ngan, Nga T. Q. Nguyen, Hoang Van Minh, Michael Donnelly, Ciaran O’Neill

**Affiliations:** 1 Centre for Public Health, Queen’s University Belfast, Belfast, UK; 2 Centre for Population Health Sciences, Hanoi University of Public Health, Hanoi, Viet Nam; 3 Department of Pharmaceutical Administration, Faculty of Pharmacy, University of Medicine and Pharmacy at Ho Chi Minh City, Ho Chi Minh City, Viet Nam

**Keywords:** Clinical breast examination, Breast cancer, Systematic reviews, LMICs

## Abstract

**Background:**

There is uncertainty about the effectiveness of clinical breast examination (CBE) and conflicting recommendations regarding its usefulness as a screening tool for breast cancer. This paper provides an overview of systematic reviews that assessed the effectiveness of CBE as a ‘stand-alone’ screening modality for breast cancer compared to no screening and focused on its value in low- and middle-income countries (LMICs).

**Methods:**

We searched MEDLINE, EMBASE, Scopus, Web of Science, and the Cochrane Database of Systematic Reviews for systematic reviews reporting the effectiveness of CBE published prior to October 29, 2019. The main outcomes assessed were mortality and down staging. The AMSTAR 2 checklist was used to assess the methodological quality of the reviews including risk of bias.

**Results:**

Eleven systematic reviews published between 1993 and 2019 were identified. There was no direct evidence that CBE reduced breast cancer mortality. Indirect evidence suggested that a well-performed CBE achieved the same effect as mammography regarding mortality despite its apparently lower sensitivity (40–69% for CBE vs 77–95% for mammography). Greater sensitivity was recorded among younger and Asian women. Moreover, CBE contributed between 17 and 47% of the shift from advanced to early stage cancer.

**Conclusions:**

CBE merits attention from health system and service planners in LMICs where a national screening programme based on mammography would be prohibitively expensive. In particular, it is likely that considerable value would be gained from conducting implementation scientific research in countries with large numbers of Asian women and/or where younger women are at higher risk.

**Registration:**

PROSPERO, registration number CRD42019126798.

**Supplementary information:**

The online version contains supplementary material available at 10.1186/s12885-020-07521-w.

## Background

Breast cancer accounted for the greatest incidence of new cases of cancer and cancer deaths among women worldwide, at 25 and 15% respectively [1]. Screening for breast cancer has been widely promoted, especially in high income countries (HICs) in order to reduce the burden of the disease [2, 3]. The effectiveness of the three most common screening modalities Mammography (MMR), Clinical breast examination (CBE), and Breast self-examination (BSE) has been assessed over a long time. However, the attention and coverage in terms of published scientific studies varies with CBE receiving the least investigative attention of the three modalities.

Surprisingly, perhaps, the effectiveness of CBE remains undetermined since the first randomised controlled trial (RCT) of breast cancer screening in 1963 [2, 4]. Different professional organizations have released conflicting guidelines/recommendations on CBE. The Canadian Task Force on Preventive Health Care (CTFPHC) in 2011 and 2018, the American Cancer Society (ACS) in 2015, and the Japan National Cancer Center (JNCC) in 2016 recommended not using CBE for population-based screening [5–8]. The U.S. Preventive Services Task Force (USPSTF) in 2009 concluded that there was insufficient evidence to recommend for or against CBE while the American College of Obstetricians and Gynaecologists in 2003 and the National Comprehensive Cancer Network in 2014 and 2019 recommended CBE every 1–3 years for women aged 25 to 39 years and annually for women aged 40+ years [9, 10]. The discord between guidelines/recommendations may arise from differences regarding the methods and quality of systematic reviews that were used to inform them, contextual variation in relation to the assessment of evidence and the adequacy of the evidence.

Most recommendations and/or systematic reviews originated in HICs where breast cancer screening has almost become synonymous with mammography [11–13]. Generally, in HICs, there is ready access to mammography whereas CBE as a ‘stand-alone’ screening modality does not appear to warrant attention or use [7]. However, mammography is expensive and less effective in women aged 40–49 years old and, therefore, it struggles to demonstrate sufficient value in low- and middle-income countries (LMICs) where resources are limited and women tend to be diagnosed at a younger age [14–16]. Indeed, the World Health Organization (WHO) does not recommend mammography for LMICs [17]. In these countries, CBE as an alternative low-cost screening modality may be more appealing [17]. RCTs and pilot studies of CBE as a screening modality conducted in Malawi, Sudan, Philippines, Egypt, and India underscore the interest of LMICs in CBE [18–23].

Recently, Mandrik et al. (2019) published the first overview of systematic reviews looking at the benefits and harms of mammography, CBE, ultrasonography, and BSE [4]. However, the overview paid little attention to CBE compared to mammography and important CBE studies were not included. Arguably, two very low-quality reviews should have been omitted from the synthesis and the overview did not contain any report of a sub-group analysis in relation to CBE. Furthermore, the overview did not summarise evidence related to down staging as an outcome of CBE, an outcome that may nevertheless be vital to the consideration of CBE as a screening modality [4].

The limitations of the mentioned overview, uncertainty about the effectiveness of CBE, conflicting recommendations from HICs related to CBE, and the diverse contexts of LMICs underscore the need for a comprehensive and critical overview of systematic reviews dedicated to CBE. In particular, there is a need to identify, describe, and appraise available evidence, changes over time, and to examine evidence regarding down staging as well as mortality within a LMIC context for whom screening based on mammography may not be economically feasible. This overview of systematic reviews reports the benefits, harms, and accuracy of CBE as a ‘stand-alone’ screening modality for breast cancer in women who are not at high-risk, compared to no screening.

## Methods

Prior to the conduct of this overview, a protocol detailing the methods was developed and registered with International Prospective Register of Systematic Reviews (PROSPERO, #CRD42019126798). The report of this overview adheres to PRISMA (Preferred Reporting Items for Systematic Reviews and Meta-Analyses) guidelines (complete PRISMA checklist is provided in Additional information).

### Search strategy and selection criteria

Selection criteria for studies were based on the PICOS framework (PICOS – Population, intervention, comparator, outcome, study type) as follows: (1) Population: women aged 18+ years without a high-risk of breast cancer; (2) Intervention: CBE; (3) Comparator: no screening or other screening modalities; (4) Outcomes included were in relation to benefits (mortality and stage of detected tumour), harms (false-positive rate, over diagnosis, and overtreatment) and accuracy (sensitivity, specificity, positive predicted value, and negative predicted value); (5) Study type: systematic review with or without meta-analysis (Details of inclusion and exclusion criteria are presented in Appendix 1, Supplement materials).

Five bibliographic databases were searched: MEDLINE (via Ovid, 1946-present), EMBASE (via Ovid, 1947-present), Scopus (2004-present), Web of Science (1900-present), and the Cochrane Database of Systematic Reviews (1992-present) in October 29, 2019. Search inquiries did not apply a time limit, but an English language-only restriction was applied. Key words such as ‘physical examination’, ‘palpation’, ‘breast neoplasms’, and ‘breast cancer’ were used in the searches (Detailed search strategies for all databases in Appendix 2, Supplement materials). Additional potential papers were retrieved from the reference lists of included studies and websites of relevant organizations such as WHO, International Agency for Research on Cancer (IARC), CTFPHC, USPSTF, and ACS. Grey literature such as published reports were included.

### Data collection and analysis

All citations resulting from the searches were imported into EndNote X8 reference manager. After removing duplicated citations, a selection process was conducted in three stages including 1) Title and abstract screening, 2) Full-text review, and 3) Quality assessment. The AMSTAR 2 tool (A MeaSurement Tool to Assess systematic Reviews) was used to ascertain the quality of eligible systematic reviews before including them in the synthesis. Overall appraisal regarding the confidence in the results of the review included four categories “high”, “moderate”, “low” and “critically low” [24]. We included reviews with a high or moderate rating. AMSTAR 2 is interlinked with PRISMA (Preferred Reporting Items for Systematic reviews and Meta-Analyses) statement published in 2009 [24]. Reviews published prior 2009, however, may receive a lower AMSTAR 2 rating due to changes in reporting requirements for systematic reviews over time. The review team considered and discussed this issue and the general lack of evidence about CBE. Our discussion led us to the decision to include reviews prior to 2009 with a low rating (due at least partly to reporting requirements rather the methodological quality per se) in order to avoid excluding potentially valuable information based on reporting as distinct from actual quality issues. However, reviews with a rating of critically low (content validity should not be relied on) were excluded (Ratings for all eligible reviews are presented in Appendix 3, Supplement materials).

A data extraction form created by the research team was pilot tested on three randomly selected included studies (~ 20% of all included studies) and refined accordingly. Relative risk of mortality reduction and downstaging effect (from advanced cancer to early stage cancer) were the primary measure for the benefits of CBE. False-positive rate was the primary measure for the harms of CBE. Accuracy of CBE were measured by sensitivity, specificity, and positive predicted value. In addition to the outcomes listed above (benefits, harms, and accuracy of CBE), extracted data also included general information on the articles’ objectives, design, search strategy, included studies, and quality assessment of the systematic reviews as well as their strengths and limitations. Decisions about which studies to include (during tittle/abstract screening and full-text review), AMSTAR rating, and data extraction were taken independently by two authors (TTN and NTQN) following the registered protocol. Disagreements were resolved by discussion with a third reviewer (CON).

Findings from included reviews were organized and presented (narrative synthesis) by pre-determined outcomes. In cases where a review was published as a peer-review article and as a published full report (grey literature), we synthesised and discussed related evidence from both sources. We also synthesised evidence from any updated reviews from their previous versions. When the results presented in a review were unclear, we examined original studies from which results were derived. Summaries of subgroup analysis in terms of age and race were provided where available.

## Results

**Fig. 1:**
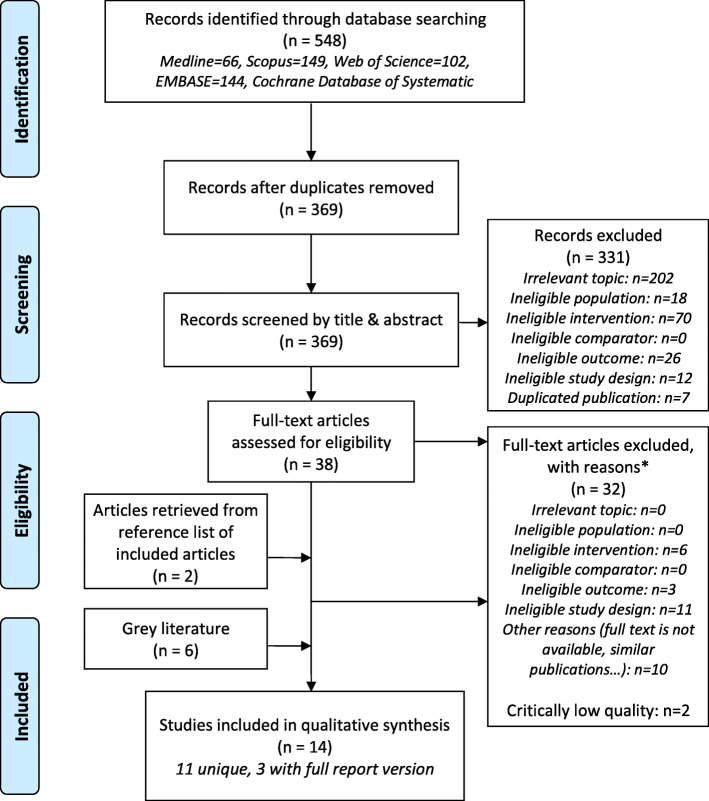
PRISMA flow diagram of literature search and selection. Reporting is in accordance to Preferred Reporting for Items for Systematic Review and Meta-Analysis (PRISMA). *Note: Topic of interest: Breast cancer screening (exclusions: other types of cancer, BC treatment, BC diagnosis); Population: Inclusions are women without a high-risk of breast cancer and never had breast cancer; Intervention: Inclusion is clinical breast examination; Comparator: Inclusions are CBE vs no screening and CBE vs other screening modalities; Outcomes: Inclusions are mortality, shift in stage of tumour at diagnosis, adverse outcomes such as false-positive results, overdiagnosis, overtreatment; Study design: Inclusions are systematic reviews and/or meta-analysis; Other reasons for exclusion: Duplicated publication (same article in different journals), full text is not available, not original article but comments, editorial notes)

We identified 548 citations from systematic searches of five databases and eight additional reviews including six grey literature reports found through manual searches of the reference lists of included citations and websites of relevant organizations (Fig. 1). After applying the inclusion/exclusion criteria, 16 potentially eligible articles/reports were identified. Two reviews [25, 26] were further excluded from the synthesis because they received the ‘critically low’ rating on AMSTAR 2 checklist (all excluded reviews with reasons for exclusion are indicated in Appendix 4, Supplement materials). The 14 included articles/reports describe 11 unique systematic reviews published in peer-reviewed journals from 1993 to 2019. There were full report versions (grey literature) of three reviews: Nelson et al. (2009), CTFPHC (2011), and Myers et al. (2015) [27–29].

Most reviews included both RCTs and non-randomised studies of interventions (NRSI) except two reviews that included only RCTs [30, 31]. Mortality as an outcome was reported in 9/11 reviews [4, 6, 7, 30, 32–36] but only one review [36] assessed down staging as an outcome. False-positive rates were addressed in 5/11 reviews [4, 33, 35–37] while 8/11 reviews reported sensitivity, specificity, and/or PPV [4, 7, 30, 32–34, 36, 37]. Summaries of reviews’ characteristics and results are presented in Table 1.

**Table 1:** Summaries of included reviews’ characteristics and results

Author (year)	Number of included studies	Focus only on CBE	AMSTAR 2^**b**^ classification	Reports on outcomes	Conclusions on CBE
	a. RCTs b. NRSI c. Systematic reviews			a. Mortality b. Downstaging c. False positive rate d. Sensitivity | Specificity | Positive predicted value	
**Fletcher SW (1993)** [30]	a. 2 b. 0 c. 0	No	Low	a. No difference in mortality rate between MMR + CBE vs CBE b. Did not report c. Did not report d. 46–64% | 99.1–99.7% | 2.9–4%	+ Mammography and CBE detect breast cancer in a complementary manner + Careful CBE may be as effective as mammography regarding mortality reduction
**Barton MB (1999)** [32]	a. 4 b. 4 c. 0	Yes	Low	a. No difference in mortality rate between MMR + CBE vs CBE b. Did not report c. Did not report d. Pooled results: 54.1% | 94% | 10.6%	+ A well-conducted CBE can detect at least 50% of asymptomatic cancers and may contribute to mortality rate reduction in women screened -- > Screening CBE should be conducted
**Humphrey LL (2002)** [33]	a. 4 b. 2 c. 1	No	Moderate	a. 14–29% mortality reduction in trials of MMR + CBE. Mortality reductions in trials of MMR + CBE were similar to trials of CBE only b. Did not report c. 13.4% d. 40–69% | 88–99% | 4–50%	+ MMR has little additive benefit in the setting of a careful, detailed CBE + No direct evidence that CBE decreases mortality
**Kosters JP (2003)** [31]	a. 1 b. 0 c. 0	No	High	a. Did not report b. Did not report c. Did not report d. Did not report	The only trial investigated CBE vs no screening was discontinued due to poor compliance -- > CBE cannot be recommended
**Elmore JG (2005)** [37]	a. 4 b. 3 c. 2	No	Low	a. Did not report b. Did not report c. 20% d. 28–54% | 94% | NR	+ CBE detects some cancers missed by MMR
**Nelson HD (2009)**^**a**^ [29, 34]	a. 4 b. 1 c. 0	No	High	a. No difference in mortality rate between MMR + CBE vs CBE (RR = 1.02, 95% CI: 0.78–1.33) b. Did not report c. Did not report d. 25.6% | NR | 1%	+ Trials of CBE are ongoing -- > no benefit on mortality has been shown at this point
**CTFPHC (2011)**^**a**^ [6, 28]	a. 4 b. 2 c. 0	No	High	a. No evidence was found to show that CBE reduced mortality due to BC or all-cause mortality b. Did not report c. Did not report d. Did not report	No evidence was found to support the benefit of CBE, either alone or in conjunction with mammography
**Myers ER (2015)**^**a**^ [27, 35]	a. 3 b. 4 c. 0	No	Moderate	a. No effect of CBE alone on mortality (based on only 1 US case-control study which also found no effect of mammography on mortality) b. Did not report c. 0.9–5.7% d. Did not report	+ Lack of evidence showing benefits of CBE alone or in conjunction with mammography + No studies assessing other critical outcomes
**Hamashima C (2016)** [7]	a. 1 b. 6 c. 1	No	Moderate	a. Based on 1 Japanese case-control study, among asymptomatic women, 1 CBE within 5 years: OR = 0.45 (95% CI: 0.22–0.89) b. Did not report c. Did not report d. 46–63% | 94.3–97.3% | NR	+ CBE is not recommended for population-based screening program due to insufficient evidence
**IARC (2016)** [36]	a. 6 b. 10 c. 1	No	Moderate	a. No difference in mortality rate between MMR + CBE vs CBE (RR = 0.97, 95% CI: 0.62–1.52) b. Mumbai trial: Significant shift to a lower stage in the screening arm compared with the control arm (RR, 1.45; 95% CI: 1.09–1.93). Kerala trial: early-stage breast cancer was 43.8% in the intervention group versus 25.4% in the control group (*P* = 0.023) c. 5.7% d. 52–85% | 93.4–96% | 1–4%	+ There is sufficient evidence that screening by CBE alone shifts the stage distribution of tumours detected towards a lower stage + There is inadequate evidence that screening by CBE alone reduces breast cancer mortality
**Mandrik O (2019)** [4]	a. 0 b. 0 c. 10	No	Moderate	a. No solid evidence of mortality reduction b. Acknowledged but did not summarise the evidence c. Higher rate of false-positive rates (did not report how higher) d. 28–36% in the community, 47–69% in RCTs in all except 1 review | > 88% in all reviews | NR	+ The review could not summarise evidence on down-staging but IARC report concluded there are sufficient evidence for this outcome + More original research on benefits and harms of CBE is required + Lack of research in LMICs -- > evidence cannot be generalized to these settings

*CBE* Clinical breast examination, *MMR* Mammography, *NR* Did not report, *NRSI* Non-randomized studies of interventions, *RCTs* Randomised controlled trials

^a^Included results from the full report version (grey literature)

^b^AMSTAR stands for A MeaSurement Tool to Assess systematic Reviews (https://amstar.ca). The AMSTAR checklist contains 16 items, of which, 7 items are marked as critical. The overall quality rating of four categories “high”, “moderate”, “low”, and “critically low” is based on the weaknesses detected in critical and non-critical items [24]

### Benefit of CBE in reducing breast cancer mortality

None of the included reviews reported any ‘direct’ evidence (evidence from RCTs that compared CBE with no screening) of a benefit from CBE on breast cancer mortality [4, 6, 7, 27–37]. However, we deemed it important to also consider ‘indirect’ evidence in order to assess fully the effects of CBE on mortality. That is, evidence that came from RCT comparing CBE with CBE + MMR, case-control studies comparing CBE with no screening, and RCT comparing CBE + MMR with no screening.

Firstly, using data from the Canadian National Breast Screening Study 2 (CNBSS-2) trial, five reviews shared the same assessment that well-performed CBE could provide the same effect on mortality reduction as mammography [30, 32–34, 36]. In the 1980 CNBSS-2 trial, the breast cancer mortality rate was similar between the intervention (19,711 women received CBE + MMR yearly) and control arm (19,694 women received CBE yearly) with relative risk (RR) = 0.97 (95% CI: 0.62–1.52) [30, 32–34, 36].

Secondly, two reviews considered evidence from case-control studies [7, 29]. USPSTF 2009 review included an US case-control study of women aged 40–65 years who had obtained a CBE in the last three to 5 years - it reported no effect of CBE alone on mortality (OR = 0.94, 95% CI: 0.79–1.12 and OR = 0.8, 95% CI: 0.59–1.08, respectively) [29]. JNCC 2016 review discussed the same US study plus another case-control study from Japan which found favourable results for CBE that one CBE within 5 years among asymptomatic women aged 30+ had a protective effect (OR = 0.45, 95% CI: 0.22–0.89) [7]. While presenting contradictory results, evidence from the Japanese study received less criticism than the US study [29].

Thirdly, using the data from the US Health Insurance Plan (HIP) trial (yearly MMR + CBE vs no screening), Barton et al. (1999) identified a 30% mortality reduction in intervention arm where 45% of cancer cases were detected by CBE alone [32]. It should be noted that at the time of this trial, mammography was not well developed compared to current practice and this underdevelopment might explain the high rate of cancer cases that were detected by CBE alone as well as the large contribution of CBE to the mortality reduction in this 1963 trial.

### Does CBE lower the stage of detected tumour?

Only one of the included reviews [36] considered down staging as an assessment outcome for the effectiveness of CBE. In this IARC-conducted review, evidence for a down staging effect came from three RCTs comparing CBE with no screening (1996 Philippines trial, 1998 Mumbai trial, and 2006 Kerala trial). The results in the IARC review were unclear and did not include results from the 2000 Cairo trial and 2010 Sudan trial. Thus, we decided to examine the original five RCTs.

**Fig. 2:**
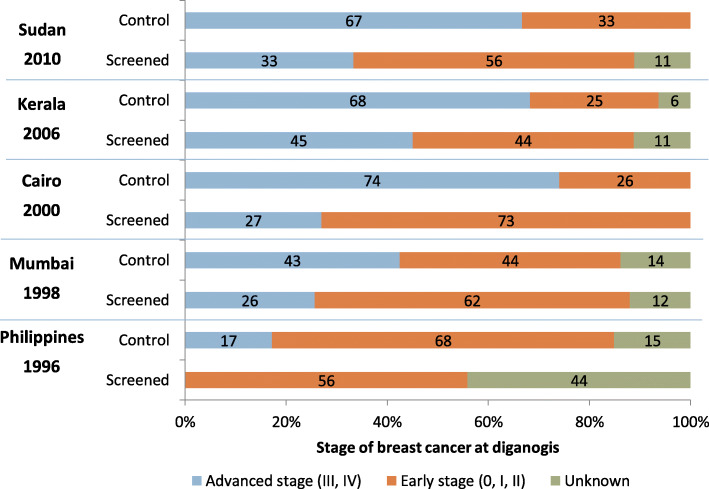
Downstaging effect of screening with clinical breast examination versus no screening, results from five randomised controlled trials [19, 20, 22, 23, 38]. *Data table reporting the frequency, percentage, risk difference, and relative risk is presented in Appendix 5, Supplement materials

With one exception (Sudan trial), all trials showed a shift to a lower stage in the detection of tumours at diagnosis that was statistically significant (Fig. 2 and Appendix 5, Supplement materials). The longest follow-up trial (three rounds of screening from 1998 to 2005 in Mumbai) reported that the relative risk of having cancer at an advanced stage in the control group compared to the screened group was 1.68 (95% CI: 1.14–2.47) [23, 36]. Similar results were observed in the Kerala trial where the RR was 1.51 (95% CI: 1.13–2.04) [22, 36]. The absolute risk difference in the proportion of advanced stage cancers (stage III & IV) was at 17% (Philippines trial) and 47% (Cairo trial) higher in the control group compared to screened group [20, 36, 38]. The Sudan trial reported an absolute risk difference of 33.4% and calculated RR at 1.20 (95% CI: 0.29–4.95) which was not statistically significant though this might have been due to the short follow-up of the trial [19].

### Harms of CBE: the False-Positive Rate (FPR)

Four reviews looked at the FPR of CBE, of which, two reported ranges from 0.9 to 5.7% in RCTs and 2.2 to 5% in cohort studies [35, 36]. The remaining two reviews used the results of one 10-year cohort study in US to report that 13.4% of women had false-positive result on CBE at least once (CBE was provided biennially) and the cumulative risk of a false-positive result after 10 CBE was 22.3% [33, 37].

### Accuracy of CBE: sensitivity, specificity, and positive predictive value

A wide range of values for the sensitivity of CBE was reported in 8/11 reviews though six of them reported a range from 40 to 69% with a pooled result of 54.1% [4, 7, 30, 32, 33]. One review reported a much higher upper boundary of 85% which came from a cohort study in Japan [36]. In contrast, another review recorded a much lower range from 28 to 36% which was derived from three cohort studies conducted in US during 1988–1998 [37]. In these studies’ setting, unlike in RCTs, the physicians (including nurses and radiologists) who performed CBE did not receive any training about the technique beforehand. Thus, quality of the procedure was questionable and might lead to the much lower sensitivity. Two reviews noted that sensitivity was higher for women in their 40s compared to women in their 50s (4–5% higher) [7, 32]. Regarding race, one review reported higher sensitivity among Asian women compared to Caucasian women (88% vs 35%, adjusted *p* = 0.04) [36]. Likewise, only one review described a difference in sensitivity when CBE was performed as a ‘stand-alone’ screening modality and when it was performed in conjunction with mammography (range: 63–69% versus 40–69%, respectively) [32].

The specificity of CBE was higher than 93% in all but one review [7, 30, 32, 36, 37]. The 2002 USPSTF review reported a slightly lower specificity of 88% based on a cohort study in US [33]. Barton et al. (1999) calculated a pooled result of 94% (95% CI: 90–97%) [32]. Sub-analysis for specificity of CBE was not reported in any included reviews.

PPV was documented in 5/11 reviews and ranged from 1 to 14% [30, 32, 34, 36]. One review reported a result of 46% which came from the 1963 HIP trial [33]. The pooled result calculated by Barton et al. (1999) was 10.6% (95% CI: 5.8–19.2) [32].

## Discussion

Since the development and wide implementation of mammography, HICs appear to have lost interest in CBE as a ‘stand-alone’ screening modality. Only one systematic review from 1999 was dedicated solely to CBE. Few reviews looked at CBE as part of screening modalities generally. The number of original studies about CBE was limited and did not increase overtime. All five RCTs that compared CBE with no screening were conducted in LMICs. These RCTs did not have any updated results and their status is unknown (except the terminated Philippine trial). Nevertheless, none of the reviews on CBE included all these RCTs. All reviews over nearly 30 years (1993–2019) on CBE regardless of publication date agreed that there was insufficient evidence to draw conclusions about the effectiveness of CBE in reducing mortality. The long-term absence of evidence on this topic clearly hinders the assessment of CBE in settings where other modalities may not be feasible. It is particularly notable that evidence regarding down staging was neglected in all but one systematic review.

### Effect on mortality

While there is no ‘direct’ evidence (from RCTs compared CBE with no screening) of an effect of CBE on mortality, the ‘indirect’ evidence (from case-control and RCTs compared CBE with other screening modalities) is indicative that CBE may be effective in reducing breast cancer mortality. CBE may have the desired effect in some settings and/or be as effective as mammography. The results illustrate the importance of context and culture. For example, Japan is the only country that has had a long-term, nation-wide population-based screening program using CBE (1987–2003) [7]. Given its longevity, studies conducted in this context may provide particularly valuable information. Included reviews reported results from a case-control study (Kanemura S, 1999) in which the odds of mortality for a group who were screened by CBE was lower than a group that did not receive screening [7]. We found similar results for a comparative analysis of age-adjusted death rates (ADR) due to breast cancer between Japanese municipalities with high- and low-coverage screening rates. Reduction in breast cancer ADR was 51.7% (*p* < 0.05) in the municipalities with coverage-rates higher than 50% compared to control municipalities (coverage < 10%) [39]. Another example is from the HIP trial which was conducted in 1960s when mammography was not well developed and thus, created an opportunity for a greater contribution from CBE to mortality reduction. A similar opportunity is also present in LMICs where mammography is absent due to inaccessibility.

### Effect on down staging

Down staging was ignored as an outcome of CBE by most systematic reviews. Given the inaccessibility of and/or unaffordability of treatments for late staged cancer that may help explain why LMICs have considerably lower survival rates compared to HICs (from 13 to 50% in LMICs compared to 80% or higher in HICs) [14, 40, 41], early detection may be particularly relevant in this context. Thus, if CBE screening can contribute significantly to the shift towards early stages of tumour at diagnosis, this outcome is promising and worthy of investigation in LMICs for whom a national screening programme based on mammography would be prohibitively expensive.

Evidence in favour of a down staging effect came from both RCTs and observational studies. In RCTs, the absolute difference in proportion of advanced-stage cancer between intervention and control group ranged from 17 to 47%. Analysis from 11 regions of Japan showed 6% difference in late stage diagnosis between patients who were found to have breast cancer by mass-screening and matched patients with breast cancer detected in out-patient clinics [42]. A 5-year pilot program in Malaysia in which community nurses performed CBE in their monthly visit to women in the community brought a 40% reduction in advanced-stage breast cancer [43]. Similarly, a 3-year pilot started in 2009 in Tanzania witnessed the difference in proportion of advanced-stage cancer between a control village and an intervention village where CBE was provided to 99% of women was 9, 23, and 17% in 2009, 2010, and 2011 respectively [44]. Evidence across these studies is consistent and comparable with a recent modelling study in which the down staging shift was assumed at 25% [45].

### Accuracy of CBE as a screening tool (sensitivity, specificity, PPV, FPR)

Systematic reviews concurred that CBE has similar specificity with mammography (at 93–97%) but lower sensitivity (40–69% vs 77–95% respectively) though it is important to note that higher sensitivity does not guarantee a reduction in mortality as can be seen from the results of CNBSS-2. In the trade-off for sensitivity, CBE has lower false-positive rate compared to mammography (1–5% for CBE vs 7–12% for mammography) [29]. Cumulative FPR after 10 year was recorded as 22.3% for CBE and as high as 50–60% for mammography. Reviews also reported that CBE sensitivity is higher in younger women (40–49 vs 50–59 years old), Asian women, and when it is applied as a stand-alone screening modality (compared to the CBE + MMR combination). Thus, in the settings where these three aspects present (younger women with a higher risk of getting BC, a large population of Asian ethnicity, and CBE can be applied only as stand-alone screening modality), CBE screening may be of particular interest.

### Strengths and limitations

This is the first overview of systematic reviews to examine the effectiveness of CBE as a stand-alone screening modality. It is only the second review that is dedicated to CBE per se (rather than spreading the investigative focus to other screening modalities such as mammography, ultrasound, and magnetic resonance imaging-MRI). Our comprehensive and systematic approach to identification, selection, appraisal, and data extraction followed the methodological guidelines by the Cochrane Handbook of Systematic Reviews [46]. Any systematic review that may have been missed is due likely to an indexing issue related to the databases (e.g. reviews that were not indexed in the category of “systematic review”) rather than oversights in the search strategy. Assessment of the quality of reviews was performed using the AMSTAR 2 checklist and the collective expertise of the review team. However, an AMSTAR rating is subjective and depends on the quality of study reporting. Another limitation is that we were unable to include studies from non-English speaking countries, especially given our emphasis on LMICs.

## Conclusions

Evidence about the effectiveness of CBE is limited. There is no ‘direct’ evidence (from RCTs which compared CBE with no screening) that CBE is effective in terms of reducing breast cancer mortality. However, the ‘indirect’ evidence suggests that a well-performed CBE may bring about the same effect as mammography regarding mortality despite its apparently lower sensitivity. With respect to the intermediate outcome of down staging, CBE contributes between 17 and 47% of the shift from advanced to early stage cancer. The results are promising and of interest for LMICs where a national screening programme based on mammography is not a realistic option. The reviewed evidence points to the existence of greater effects among younger women and Asian women. Research into its value among countries with large numbers of Asian women and/or where younger women are at higher risk may be particularly valuable. While CBE may be effective, further work is required to assess the cost-effectiveness of CBE screening including factors associated with the acceptability and uptake of programs in LMICs.

## Supplementary information

**Additional file 1: Appendix 1**. Detailed inclusion and exclusion criteria; **Appendix 2**: Detailed search strategies for all databases; **Appendix 3**: Appraisal of eligible systematic reviews by AMSTAR 2 checklist; **Appendix 4**: List of excluded reviews and justification for the exclusions; **Appendix 5**: Data table for downstaging effect of screening with clinical breast examination versus no screening (Results from five randomised controlled trials-RCTs). (PDF 413 kb)

**Additional file 2: **Complete PRISMA checklist***.*** The PRISMA checklist was completed in full with section, page number, and line number of the paper which reports the information that meets the criteria of the checklist. (PDF 178 kb)

## Data Availability

The protocol of current study are available in PROSPERO repository, https://www.crd.york.ac.uk/prospero/display_record.php?RecordID=126798. Search strategies needed to replicate the study are included in the supplement materials file.

## Abbreviations

ACS: American Cancer Society
AMSTAR: A MeaSurement Tool to Assess systematic Reviews
BSE: Breast self-examination
CBE: Clinical breast examination
CI: Confidence interval
CNBSS-2: Canadian National Breast Screening Study 2
CTFPHC: Canadian Task Force on Preventive Health Care
FPR: False-positive rate
HICs: Hight income countries
HIP: US Health Insurance Plan trial
IARC: International Agency for Research on Cancer
JNCC: Japan National Cancer Center
LMICs: Low- and middle-income countries
MMR: Mammography
OR: Odds ratio
PICOS: Population – Intervention – Comparator – Outcome - Study type
PPV: Positive predictive value
PRISMA: Preferred Reporting Items for Systematic reviews and Meta-Analyses
PROSPERO: International Prospective Register of Systematic Reviews
RCTs: Randomised controlled trials
RR: Relative risk
US: United States of America
USPSTF: U.S. Preventive Services Task Force
WHO: World Health Organization
