# Supplementary material for: Effectiveness of clinical breast examination as a ‘stand-alone’ screening modality: an overview of systematic reviews
Source: BMC Cancer. 2020 Nov 9;20:1070. doi: 10.1186/s12885-020-07521-w (PMC7653771; doi:10.1186/s12885-020-07521-w)
Supplement: Supplementary file 1 — Additional file 1: Appendix 1. Detailed inclusion and exclusion criteria; Appendix 2: Detailed search strategies for all databases; Appendix 3: Appraisal of eligible systematic reviews by AMSTAR 2 checklist; Appendix 4: List of excluded reviews and justification for the exclusions; Appendix 5: Data table for downstaging effect of screening with clinical breast examination versus no screening (Results from five randomised controlled trials-RCTs). (PDF 413 kb) [file 12885_2020_7521_MOESM1_ESM.pdf]

Appendix 1: Detailed inclusion and exclusion criteria

Study	Inclusion criteria	Exclusion criteria
**Population**	• Women aged 18 and older<br>• Without a high-risk of breast cancer based on family history *(first-degree blood relatives or second-degree blood relatives had breast cancer and/or ovarian cancer)* or personal risk factors *(faulty BRCA1, BRCA2 or TP53 gene)*	• Non-human subjects<br>• Men with breast cancer<br>• Women with pre-existing or personal history of breast cancer<br>• Women considered to be at high-risk for breast cancer
**Interventions**	• Clinical breast examination as a stand-alone screening strategy<br>• Clinical breast examination in combination with other screening techniques such as mammography (film, digital, or tomosyntheis-3D mammography), magnetic resonance imaging (MRI); ultrasound; breast self-examination	• Combination screening modalities without the presence of clinical breast examination
**Comparators**	• CBE vs no screening<br>• CBE vs other screening modalities	No comparisons or outcomes of interest between:<br>• CBE vs no screening<br>• CBE vs other screening modalities
**Outcomes**	**Primary main outcomes (benefits)**<br>• Mortality (Breast cancer related mortality and all-cause mortality)<br>• Life expectancy (life-years gained by screened by screening)<br>• Quality of life (quality-adjusted life years gained by screening)<br>• Stage distribution of detected tumour (at diagnosis)<br><br>**Secondary main outcomes (harms)***<br>• False-positive results<br>• Overdiagnosis<br>• Overtreatment<br>• Emotional impacts (anxiety, depression etc.)<br><br>**Other outcomes**<br>• Sensitivity and specificity<br>• Positive predicted value (PPV), negative predicted value (NPV)	• Outcomes not listed
**Study design**	• Systematic review with or without meta-analysis	• Any other study designs (e.g. scoping reviews, narrative reviews, original studies etc.)<br>• Not a research study (e.g.: editorial, letter to the editor etc.)
**Study context**	• Any countries	• None (No restriction)
**Publication type**	• Published at anytime<br>• English language only<br>• Peer-reviewed articles and grey literature as in form of published report	• None (no time restriction)<br>• Non-English articles<br>• Personal communication and other types of grey literature (e.g.: unpublished reports, presentations etc.)

** Glossary of terms*

- *False-positive test result: A test result that indicates that a person has cancer when the person actually does not have the disease*
- *Overdiagnosis: Finding cases of cancer with a screening test (such as a mammogram or PSA test) that will never cause any symptoms. These cancers may just stop growing or go away on their own. Some of the harms caused by overdiagnosis are anxiety and having treatments that are not needed. Different from false-positive as there is a diagnosis.*
- *Overtreatment: Treatment of a cancer that would have gone away on its own or never caused any symptoms. These cancers are usually found on a screening test. Overtreatment may lead to problems and harmful side effects from cancer therapies that are not needed.*

Appendix 2: Detailed search strategies for all databases

Cochrane Database of Systematic Reviews: Simple search using the term “breast examination”

EMBASE (via Ovid)

1. breast cancer/ or breast tumour/ or basal like breast cancer/ or breast cancer molecular subtype/ or breast carcinogenesis/ or breast carcinoma/ or breast sarcoma/ or inflammatory breast cancer/ or metastatic breast cancer/ or phyllodes tumor/<br>2. (breast* adj5 (cancer* or carcinoma* or malignan* or neoplasm* or tumo?r*)).ti,ab.<br>3. breast examination/ or breast self examination/<br>4. physical examination/ or palpation/<br>5. gynecological examination/<br>6. "clinical breast examination*".ti,ab.<br>7. "self exam*".ti,ab.<br>8. self examination/<br>9. 1 or 2<br>10. 3 or 4 or 5 or 6 or 7 or 8<br>11. 9 and 10<br>12. limit 11 to (meta analysis or "systematic review")<br>13. limit 12 to english language

MEDLINE (via Ovid)

1. breast neoplasms/ or breast carcinoma in situ/ or carcinoma, ductal, breast/ or carcinoma, lobular/ or "hereditary breast and ovarian cancer syndrome"/ or inflammatory breast neoplasms/ or unilateral breast neoplasms/ or triple negative breast neoplasms/<br>2. (breast* adj5 (cancer* or carcinoma* or malignan* or neoplasm* or tumo?r*)).ti,ab.<br>3. Breast Self-Examination/<br>4. Physical Examination/<br>5. Palpation/<br>6. Gynecological Examination/<br>7. "clinical breast examination*".ti,ab.<br>8. "self exam*".ti,ab.<br>9. Self-Examination/<br>10. 1 or 2<br>11. 3 or 4 or 5 or 6 or 7 or 8 or 9<br>12. 10 and 11<br>13. limit 12 to (meta analysis or systematic reviews)<br>14. limit 13 to english language

Scopus

TITLE-ABS-KEY (breast* W/5 (cancer* OR carcinoma* OR malignan* OR neoplasm* OR tumo*r*))) AND (TITLE-ABS-KEY (self-exam* OR "breast *exam*" OR "physical exam*" OR "gyn*ecological exam*" OR palpat*)) AND (TITLE-ABS-KEY (meta*analysis* OR "systematic review*")) AND (LIMIT-TO (LANGUAGE , "English"))

Web of Science

#1	**TOPIC:** (breast* near/5 (cancer* or carcinoma* or malignan* or neoplasm* or tumo$r*))<br>*DocType=All document types; Language=All languages;*
#2	TS=(self-exam* or "breast *exam*" or "physical exam*" or "gyn$ecological exam*" or palpat*)<br>*DocType=All document types; Language=All languages;*
#3	#2 AND #1<br>*DocType=All document types; Language=All languages;*
#4	**TOPIC:** (meta$analysis* or "systematic review*")<br>*DocType=All document types; Language=All languages;*
#5	#4 AND #3<br>*DocType=All document types; Language=All languages;*
#6	#4 AND #3<br>Refined by: **LANGUAGES:** (ENGLISH)<br>*DocType=All document types; Language=All languages;*

Appendix 3: Appraisal of eligible systematic reviews by AMSTAR 2 checklist

Systematic review	AMSTAR 2 overall rating†	16 items in AMSTAR 2 checklist															
		Inclusion of PICO in research questions & inclusion criteria	Protocol*	Study design inclusion criteria	Comprehensive search strategy*	Study selection in duplicate	Data extraction in duplicate	List of excluded studies & justification*	Description of included studies	Risk of bias (ROB) assessment	Source of funding	Meta-analysis*	Impacts of ROB in individual studies	Impacts of ROB on results*	Discussion on heterogeneity	Publication bias*	Conflict of interest
Fletcher SW (1993) [1]	LOW	Y	N	Y	N	Y	Y	N	Y	Y	NA	NA	NA	Y	Y	NA	N
Kerlikowske K (1995) ‡ [2]	CRITICALLY LOW	Y	N	Y	N	N	Y	N	Y	N	Y	NA	NA	N	N	NA	N
Barton MB (1999) [3]	LOW	Y	N	Y	Y	Y	N	N	Y	N	Y	Y	N	N	N	N	N
Humphrey LL (2002) [4] (USPSTF)	Moderate	Y	N	N	Y	N	Y	Y	Y	Y	Y	NA	NA	Y	Y	NA	N
Green BB (2003) ‡ [5]	CRITICALLY LOW	Y	N	N	N	N	N	N	Y	N	N	NA	NA	N	N	NA	N
Kosters JP (2003) [6]	HIGH	Y	Y	N	Y	Y	Y	Y	Y	Y	Y	NA	NA	Y	Y	NA	Y
Elmore JG (2005) [7]	LOW	Y	N	Y	Y	N	N	N	Y	N	Y	NA	NA	N	Y	NA	Y
Nelson HD (2009) (published article and full report for USPSTF) [8,9]	HIGH	Y	p.Y	Y	Y	N	Y	Y	Y	Y	Y	NA	NA	Y	Y	NA	Y
CTFPHC (2011) (published article and full report) [10,11]	HIGH	Y	p.Y	Y	Y	Y	Y	Y	Y	Y	Y	NA	NA	Y	Y	NA	Y
Myers ER (2015) (published article and full report for ACS) [12,13]	Moderate	Y	p.Y	Y	p.Y	Y	Y	Y	Y	Y	Y	NA	NA	Y	Y	NA	Y
Hamashima C (2016) [14]	Moderate	Y	Y	Y	Y	Y	N	N	p.Y	Y	Y	NA	NA	Y	Y	NA	Y
IARC (2016) [15]	Moderate	Y	Y	Y	p.Y	Y	Y	N	Y	Y	Y	NA	NA	Y	Y	NA	Y
Mandrik O (2019) [16]	Moderate	Y	Y	Y	Y	Y	Y	Y	N	Y	Y	NA	NA	Y	Y	NA	Y

** Items are usually regarded as critical within AMSTAR 2 checklist (but not always and can be dismissed and/or substitute in certain systematic reviews)*

† Assessment used the full report (where available) and considered only the part related to CBE. Rating is classified as 'High' (no or one non-critical weakness), 'Moderate' (More than one non-critical weakness), 'Low' (One critical flaw with or without non-critical weakness), and 'Critically low' (More than one critical weakness with or without non-critical weakness)

‡ Excluded in analysis due to the critically low quality of the report

ACS: American Cancer Society | CTFPHC: Canadian Task Force on Preventive Health Care | IARC: International Agency for Research on Cancer | NA: not applicable | N: No| PICO: Population, Intervention, Comparator, Outcome | p.Y: partial Yes | ROB: Risk of bias | USPSTF: US Preventive Services Task Force | Y: Yes

Appendix 4: List of excluded reviews and justification for the exclusions

This list includes all potentially relevant reviews (that are eligible for full-text assessment) with justification for the exclusion of each. The list does not contain articles excluded due to duplicate or during the title and abstract screening.

Ineligible population: None was excluded due to this reason

Ineligible intervention: Six reviews were excluded

1. Cutler W, Burki R, Kolter J, Chambliss C, Friedmann E, Hart K. Invasive Breast Cancer Incidence in 2,305,427 Screened Asymptomatic Women: Estimated Long Term Outcomes during Menopause Using a Systematic Review. *PloS one* 2015; **10**(6): e0128895.
2. Huggenberger IK, Andersen JS. Predictive value of the official cancer alarm symptoms in general practice - a systematic review. *Danish Medical Journal* 2015; **62**(5).
3. Kolak A, Kaminska M, Sygit K, et al. Primary and secondary prevention of breast cancer. *Annals of Agricultural and Environmental Medicine* 2017; **24**(4): 549-53.
4. Mettlin C, Murphy GP. Breast cancer screening in premenopausal women: Current recommendations and opportunities for research. *Annals of Medicine* 1995; **27**(4): 461-5.
5. Mettlin C, Smart CR. Breast cancer detection guidelines for women aged 40 to 49 years: Rationale for the American Cancer Society reaffirmation of recommendations. *Ca-A Cancer Journal for Clinicians* 1994; **44**(4): 248-55.
6. Wald N, Chamberlain J, Hackshaw A. Consensus Conference on Breast Cancer Screening: Paris, February 4-5, 1993 - Report of the Evaluation Committee. *Oncology* 1994; **51**(4): 380-9.

Ineligible comparator: None was excluded due to this reason

Ineligible outcome: Three reviews were excluded

7. Molina Y, Thompson B, Espinoza N, Ceballos R. Breast cancer interventions serving US-based Latinas: Current approaches and directions. *Women's Health* 2013; **9**(4): 335-50.
8. Zagouri F, Liakou P, Bartsch R, et al. Discrepancies between ESMO and NCCN breast cancer guidelines: An appraisal. *Breast* 2015; **24**(4): 513-23.
9. Zelle SG, Baltussen RM. Economic analyses of breast cancer control in low- and middle-income countries: A systematic review. *Systematic Reviews* 2013; **2**(1): 20.

Ineligible study design: Eleven reviews were excluded

10. Al-Foheidi M, Al-Mansour MM, Ibrahim EM. Breast cancer screening: Review of benefits and harms, and recommendations for developing and low-income countries. *Medical Oncology* 2013; **30**(2).
11. Berg AO, Allan JD, Frame PS, et al. Screening for breast cancer: Recommendations and rationale. *Annals of Internal Medicine* 2002; **137**(5 I): 344-6.
12. Brodersen J, Jorgensen KJ, Gotzsche PC. The benefits and harms of screening for cancer with a focus on breast screening. *Polskie Archiwum Medycyny Wewnetrznej-Polish Archives of Internal Medicine* 2010; **120**(3): 89-93.
13. Ford K, Marcus E, Lum B. Breast cancer screening, diagnosis, and treatment. *Disease-a-Month* 1999; **45**(9): 337-405.
14. Harris R. Effectiveness: The next question for breast cancer screening. *Journal of the National Cancer Institute* 2005; **97**(14): 1021-3.
15. Jatoi I, Anderson WF. In brief. *Current Problems in Surgery* 2005; **42**(9): 616-8.
16. Jatoi I, Anderson WF. Cancer screening. *Current Problems in Surgery* 2005; **42**(9): 620-82.
17. Law M. Screening without evidence of efficacy. *British Medical Journal* 2004; **328**(7435): 301-2.
18. Marilyn Leitch A, Dodd GD, Costanza M, et al. American Cancer Society guidelines for the early detection of breast cancer: Update 1997. *Ca-A Cancer Journal for Clinicians* 1997; **47**(3): 150-3.
19. Miser WF. Cancer Screening in the Primary Care Setting. The Role of the Primary Care Physician in Screening for Breast, Cervical, Colorectal, Lung, Ovarian, and Prostate Cancers. *Primary Care - Clinics in Office Practice* 2007; **34**(1): 137-67.
20. Rodger A. The perspective of half a century: How breast cancer care has changed or has it? *Breast* 2006; **15**(6): 691-2.

Other reasons (full text is not available, similar publications…): Ten reviews were excluded

21. Balducci L. Cancer and age. *FORUM - Trends in Experimental and Clinical Medicine* 1994; **4**(5): 554-65.	Full-text is not available
22. Costanza ME, Edmiston KL. Breast cancer screening: Early recognition. *Comprehensive Therapy* 1997; **23**(1): 7-12.	Full-text is not available
23. Lee H-B, Han W. Unique features of young age breast cancer and its management. *Journal of breast cancer* 2014; **17**(4): 301-7.	Not in English (except the tables)
24. Mohil D, Banerjee M, Mankotia D, et al. Cancer: An insight into the detection, assessment and treatment. *Asian Journal of Pharmaceutical and Clinical Research* 2018; **11**(3): 124.	Not full-text publication (conference abstract)
25. Oeffinger KC, Fontham ETH, Etzioni R, et al. Breast cancer screening for women at average risk: 2015 Guideline update from the American cancer society. *JAMA - Journal of the American Medical Association* 2015; **314**(15): 1599-614.	Duplicate with other article
26. Paulsen C, Gebhardt S. An evidence-based breast cancer screening protocol for O&G. *Obstetrics and Gynaecology Forum* 2019; **29**(1): 17-20.	Full-text is not available
27. Petitti DB, Calonge N, LeFevre ML, Melnyk BM, Wilt TJ, Sanford Schwartz J. Breast cancer screening: From science to recommendation. *Radiology* 2010; **256**(1): 8-14.	Duplicate with other article
28. Rosegger V, Augesky-Stocker D, Hurkmans E. Reliability of skin and subcutaneous tissue assessments: A systematic review. *Physiotherapy (United Kingdom)* 2015; **101**(SUPPL. 1): eS616-eS7.	Not full-text publication (conference abstract)
29. The Canadian Task Force on Preventive Health Care. Breast cancer screening: Part A. An evidence report to inform an update of the Canadian Task Force on Preventive Health Care 2011 Guideline Ontario, Canada: Knowledge Synthesis Group, Ottawa Methods Centre, Ottawa Hospital Research Institute, 2017.	Updated version with no new information related to clinical breast examination
30. Nelson HD, Tyne K, Naik A, et al. Screening for Breast Cancer: A Systematic Review to Update the 2009 U.S. Preventive Services Task Force Recommendation. Rockville, MD: Agency for Healthcare Research and Quality, 2016.	Updated version with no new information related to clinical breast examination

'Critically low' rating based on AMSTAR 2 checklist: Two reviews were excluded

31. Green BB, Taplin SH. Breast cancer screening controversies. *Journal of the American Board of Family Practice* 2003; **16**(3): 233-41.
32. Kerlikowske K, Grady D, Rubin SM, Sandrock C, Ernster VL. Efficacy of screening mammography. A meta-analysis. *JAMA* 1995; **273**(2): 149-54.

Appendix 5: Data table for downstaging effect of screening with clinical breast examination versus no screening (Results from five randomised controlled trials-RCTs)

RCTs comparing CBE vs no screening	Screened n (%)			Control n (%)			Risk difference in advanced cancer	RR (advanced cancer, control vs screen)
	Total	Early	Advanced	Total	Early	Advanced		
**1996 Philippines trial** [17]	34	19 (55.9)	0 (0)	99	67 (67.7)	17 (17.2)	17.2%	..*
**1998 Mumbai trial** [18]	125	78 (62.4)	32 (25.6)	87	38 (43.7)	37 (42.5)	16.9%	1.68* (1.14-2.47)
**2000 Cairo trial†** [19]		(73)	(27)		(26)	(74)	47%	2.74*
**2006 Kerala trial** [20]	80	35 (43.8)	36 (45.0)	63	16 (25.4)	43 (68.3)	23.3%	1.51* (1.13-2.04)
**2010 Sudan trial** [21]	9	5 (55.6)	3 (33.3)	3	1 (33.3)	2 (66.7)	33.4%	1.20 (0.29-4.95)

** Significant difference*

† Data on frequency is not available

All presented data was preliminary results after the first round of screening except data of Mumbai trial which was interim results after three rounds of screening (7-year of follow-up). Philippines trial was terminated after first screening round due to poor compliance with the follow-up of screen-positive women.

Reference (for appendix 1-5)

1. Fletcher SW, Black W, Harris R, Rimer BK, Shapiro S. Report of the International Workshop on Screening for Breast Cancer. *Journal of the National Cancer Institute* 1993; **85**(20): 1644-56.
2. Kerlikowske K, Grady D, Rubin SM, Sandrock C, Ernster VL. Efficacy of screening mammography. A meta-analysis. *JAMA* 1995; **273**(2): 149-54.
3. Barton MB, Harris R, Fletcher SW. The rational clinical examination. Does this patient have breast cancer? The screening clinical breast examination: should it be done? How? *JAMA* 1999; **282**(13): 1270-80.
4. Humphrey LL, Helfand M, Chan BKS, Woolf SH. Breast cancer screening: A summary of the evidence for the U.S. Preventive Services Task Force. *Annals of internal medicine* 2002; **137**(5 I): 347-60.
5. Green BB, Taplin SH. Breast cancer screening controversies. *Journal of the American Board of Family Practice* 2003; **16**(3): 233-41.
6. Kosters JP, Gotzsche PC. Regular self-examination or clinical examination for early detection of breast cancer. *The Cochrane database of systematic reviews* 2003; (2): CD003373.
7. Elmore JG, Armstrong K, Lehman CD, Fletcher SW. Screening for breast cancer. *Journal of the American Medical Association* 2005; **293**(10): 1245-56.
8. Nelson HD, Tyne K, Naik A, et al. Screening for Breast Cancer: Systematic Evidence Review Update for the U. S. Preventive Services Task Force. Rockville, MD: Agency for Healthcare Research and Quality, 2009.
9. Nelson HD, Tyne K, Naik A, et al. Screening for breast cancer: an update for the U.S. Preventive Services Task Force. *Annals of internal medicine* 2009; **151**(10): 727-42.
10. Fitzpatrick-Lewis D, Hodgson N, Ciliska D, Peirson L, Gauld M, Liu YY. Breast Cancer Screening. Ontario, Canada: McMaster University, 2011.
11. The Canadian Task Force on Preventive Health Care. Recommendations on screening for breast cancer in average-risk women aged 40-74 years. *CMAJ* 2011; **183**(17): 1991-2001.
12. Duke Evidence Synthesis Group. Systematic Review of Cancer Screening Literature for Updating American Cancer Society Breast Cancer Screening Guidelines, 2014.
13. Myers ER, Moorman P, Gierisch JM, et al. Benefits and Harms of Breast Cancer Screening: A Systematic Review. *JAMA* 2015; **314**(15): 1615-34.
14. Hamashima C, Hattori M, Honjo S, et al. The Japanese guidelines for breast cancer screening. *Japanese journal of clinical oncology* 2016; **46**(5): 482-92.
15. International Agency for Research on Cancer Working Group on the Evaluation of Cancer-Preventive Interventions. IARC Handbooks of Cancer Prevention - Volume 15: Breast Cancer Screening. Lyon, France: International Agency for Research on Cancer (IARC), 2016.
16. Mandrik O, Zielonke N, Meheus F, et al. Systematic reviews as a 'lens of evidence': Determinants of benefits and harms of breast cancer screening. *International journal of cancer* 2019; **145**(4): 994-1006.
17. Pisani P, Parkin DM, Ngelangel C, et al. Outcome of screening by clinical examination of the breast in a trial in the Philippines. *International journal of cancer* 2006; **118**(1): 149-54.
18. Mittra I, Mishra GA, Singh S, et al. A cluster randomized, controlled trial of breast and cervix cancer screening in Mumbai, India: methodology and interim results after three rounds of screening. *International journal of cancer* 2010; **126**(4): 976-84.
19. Miller AB. Practical Applications for Clinical Breast Examination (CBE) and Breast Self-Examination (BSE) in Screening and Early Detection of Breast Cancer. *Breast Care (Basel)* 2008; **3**(1): 17-20.
20. Sankaranarayanan R, Ramadas K, Thara S, et al. Clinical breast examination: preliminary results from a cluster randomized controlled trial in India. *J Natl Cancer Inst* 2011; **103**(19): 1476-80.
21. Abuidris DO, Elsheikh A, Ali M, et al. Breast-cancer screening with trained volunteers in a rural area of Sudan: a pilot study. *The Lancet Oncology* 2013; **14**(4): 363-70.
