# Supplementary material for: Effectiveness of clinical breast examination as a ‘stand-alone’ screening modality: an overview of systematic reviews
Source: BMC Cancer. 2020 Nov 9;20:1070. doi: 10.1186/s12885-020-07521-w (PMC7653771; doi:10.1186/s12885-020-07521-w)
Supplement: Supplementary file 2 — Additional file 2: Complete PRISMA checklist. The PRISMA checklist was completed in full with section, page number, and line number of the paper which reports the information that meets the criteria of the checklist. (PDF 178 kb) [file 12885_2020_7521_MOESM2_ESM.pdf]

PRISMA 2009 Checklist

Additional information for article "Effectiveness of clinical breast examination as a 'stand-alone' screening modality: An overview of systematic reviews"

Section/topic	#	Checklist item	Reported on page #
**TITLE**			
Title	1	Identify the report as a systematic review, meta-analysis, or both.	Page 2, line 15-16
**ABSTRACT**			
Structured summary	2	Provide a structured summary including, as applicable: background; objectives; data sources; study eligibility criteria, participants, and interventions; study appraisal and synthesis methods; results; limitations; conclusions and implications of key findings; systematic review registration number.	Page 2, line 17-38
**INTRODUCTION**			
Rationale	3	Describe the rationale for the review in the context of what is already known.	Background section, page 3-4, line 41-91
Objectives	4	Provide an explicit statement of questions being addressed with reference to participants, interventions, comparisons, outcomes, and study design (PICOS).	Background section, page 4-5, line 89-91<br>Methods section, page 5, line 99-106 (PICOS)
**METHODS**			
Protocol and registration	5	Indicate if a review protocol exists, if and where it can be accessed (e.g., Web address), and, if available, provide registration information including registration number.	Methods section, page 5, line 93-95<br>Link to protocol was provided at page 16, line 370
Eligibility criteria	6	Specify study characteristics (e.g., PICOS, length of follow-up) and report characteristics (e.g., years considered, language, publication status) used as criteria for eligibility, giving rationale.	Methods section, page 5, line 99-106 and 109-113<br>Appendix 1, Supplement materials (Detailed inclusion and exclusion criteria)
Information sources	7	Describe all information sources (e.g., databases with dates of coverage, contact with study authors to identify additional studies) in the search and date last searched.	Methods section, page 5, line 107-109 and 113-116
Search	8	Present full electronic search strategy for at least one database, including any limits used, such that it could be repeated.	Methods section, page 5, line 109-113<br>Appendix 2, Supplement materials (Detailed search

PRISMA 2009 Checklist

Section/topic	#	Checklist item	Reported on page #
			strategies for all databases)
Study selection	9	State the process for selecting studies (i.e., screening, eligibility, included in systematic review, and, if applicable, included in the meta-analysis).	Methods section, page 6, line 118-120, 129-134, 143-146, and Figure 1
Data collection process	10	Describe method of data extraction from reports (e.g., piloted forms, independently, in duplicate) and any processes for obtaining and confirming data from investigators.	Methods section, page 6, line 135-136 and 143-146
Data items	11	List and define all variables for which data were sought (e.g., PICOS, funding sources) and any assumptions and simplifications made.	Methods section, page 7, line 136-143
Risk of bias in individual studies	12	Describe methods used for assessing risk of bias of individual studies (including specification of whether this was done at the study or outcome level), and how this information is to be used in any data synthesis.	Methods section, page 6, line 120-134
Summary measures	13	State the principal summary measures (e.g., risk ratio, difference in means).	Methods section, page 6-7, line 136-140
Synthesis of results	14	Describe the methods of handling data and combining results of studies, if done, including measures of consistency (e.g., I^2) for each meta-analysis.	Methods section, page 7, line 147-153
Risk of bias across studies	15	Specify any assessment of risk of bias that may affect the cumulative evidence (e.g., publication bias, selective reporting within studies).	N/A
Additional analyses	16	Describe methods of additional analyses (e.g., sensitivity or subgroup analyses, meta-regression), if done, indicating which were pre-specified.	N/A
**RESULTS**			
Study selection	17	Give numbers of studies screened, assessed for eligibility, and included in the review, with reasons for exclusions at each stage, ideally with a flow diagram.	Results section, page 7, line 155-164 and Figure 1 (Flow diagram of study selection process)
Study characteristics	18	For each study, present characteristics for which data were extracted (e.g., study size, PICOS, follow-up period) and provide the citations.	Results section, page 8, line 166-171<br>Table 1, page 22-24 (Summaries of reviews' characteristics and results)
Risk of bias within studies	19	Present data on risk of bias of each study and, if available, any outcome level assessment (see item 12).	Table 1, page 22-24<br>Appendix 3, Supplement materials (AMSTAR rating for all eligible reviews)

PRISMA 2009 Checklist

Section/topic	#	Checklist item	Reported on page #
Results of individual studies	20	For all outcomes considered (benefits or harms), present, for each study: (a) simple summary data for each intervention group (b) effect estimates and confidence intervals, ideally with a forest plot.	Table 1, page 22-24
Synthesis of results	21	Present results of each meta-analysis done, including confidence intervals and measures of consistency.	Results section, page 8-11, line 173-246
Risk of bias across studies	22	Present results of any assessment of risk of bias across studies (see Item 15).	N/A
Additional analysis	23	Give results of additional analyses, if done (e.g., sensitivity or subgroup analyses, meta-regression [see Item 16]).	N/A
**DISCUSSION**			
Summary of evidence	24	Summarize the main findings including the strength of evidence for each main outcome; consider their relevance to key groups (e.g., healthcare providers, users, and policy makers).	Discussions section, page 11-14, line 248-310
Limitations	25	Discuss limitations at study and outcome level (e.g., risk of bias), and at review-level (e.g., incomplete retrieval of identified research, reporting bias).	Discussions section, page 14, line 311-324
Conclusions	26	Provide a general interpretation of the results in the context of other evidence, and implications for future research.	Conclusions section, page 14, line 327-339
**FUNDING**			
Funding	27	Describe sources of funding for the systematic review and other support (e.g., supply of data); role of funders for the systematic review.	Declarations section, page 16, line 373-378

From: Moher D, Liberati A, Tetzlaff J, Altman DG, The PRISMA Group (2009). Preferred Reporting Items for Systematic Reviews and Meta-Analyses: The PRISMA Statement. PLoS Med 6(7): e1000097. doi:10.1371/journal.pmed1000097

For more information, visit: **www.prisma-statement.org**.
